# Supplementary material for: Clinico-pathological and treatment characteristics of HIV and non-HIV related vulvar cancers: Analysis of a South African cohort
Source: PLoS One. 2025 Jul 1;20(7):e0327119. doi: 10.1371/journal.pone.0327119 (PMC12212490; doi:10.1371/journal.pone.0327119)
Supplement: Attachment — Submitted filename: PONE-D-24-50571_reviewer edits P.B12FEB2025.pdf [file pone.0327119.s002.pdf]

PLOS ONE

Clinico-pathological and treatment characteristics of HIV and non-HIV related vulvar cancers: Analysis of a South African cohort

--Manuscript Draft--

**Manuscript Number:**	PONE-D-24-50571
**Article Type:**	Research Article
**Full Title:**	Clinico-pathological and treatment characteristics of HIV and non-HIV related vulvar cancers: Analysis of a South African cohort
**Short Title:**	Clinico-pathologic characteristics of HIV and non-HIV related vulvar cancers: A South African audit
**Corresponding Author:**	Adekunle Emmanuel Sajo, MB;BS, MPhil<br>University of Pretoria Faculty of Health Sciences<br>Pretoria, Gauteng SOUTH AFRICA
**Keywords:**	Vulvar cancer; HIV; treatment
**Abstract:**	Objective<br>The main objective of this study is to review the clinical, pathological and treatment characteristics of patients with vulvar cancer presenting to Steve Biko Academic Hospital. This includes patients who have received surgery and/or radiotherapy as part of their treatment.<br>Material and Methods<br>A retrospective review of clinical case records of patients presenting to the Gynaecology Oncology and Radiation Oncology departments of the hospital. The clinical, pathology and radiotherapy treatment records of patients presenting between January 2012 and December 2022 were reviewed for this study<br>Results<br>317 vulvar cancer patients between 2012 and 2022 were included in the analysis. The average age was 45.1±12.7. Forty percent of the participants were younger than 40 years. More than 75% of them were women living with HIV and were all on antiretroviral treatment. The average age of those who were HIV positive was 20 years lower than their HIV negative counterparts, $p < 0.0001$. Their mean haemoglobin (Hb) at presentation was 10.7g/dL. Squamous cell carcinoma was the most common histological type in 96.5% of cases. Sixty four percent of the cases presented in advanced stage. About 48% of those who had primary radiation received curative doses. The median time to completion of radiotherapy treatment was higher among those who received primary radiotherapy as compared to those who received adjuvant treatment, 51.5 vs 46 days, p 0.039. The annual average age decreased from 56 years to as low as 40 years, a mean difference of 16 years, p 0.012.<br>Conclusion<br>It is striking that vulvar cancer is no longer a disease of elderly women. Its incidence is now high among women below 50 years. The study also showed an upward trend in the number of vulvar cancer cases in contrast to the declining average age at diagnosis. There is need for more vulvo-perineal surveillance of HIV positive women to detect early stage of vulvar cancers.
**Order of Authors:**	Adekunle Emmanuel Sajo, MB;BS, MPhil
	Adekunle Emmanuel Sajo, MB;BS, MPhil
	Edwin Mnisi, MB;ChB, MPhil, FCOG
	Sheynaz Bassa, FC Rad Oncol
	Cathy Visser, MSc
	Piet Becker, PhD
	Greta Dreyer, PhD
**Additional Information:**	
**Question**	**Response**

Powered by Editorial Manager® and ProduXion Manager® from Aries Systems Corporation

**Financial Disclosure**<br><br>Enter a financial disclosure statement that describes the sources of funding for the work included in this submission. Review the submission guidelines for detailed requirements. View published research articles from *PLOS ONE* for specific examples.<br><br>This statement is required for submission and **will appear in the published article** if the submission is accepted. Please make sure it is accurate.<br><br>**Funded studies**<br>Enter a statement with the following details:<br>• Initials of the authors who received each award<br>• Grant numbers awarded to each author<br>• The full name of each funder<br>• URL of each funder website<br>• Did the sponsors or funders play any role in the study design, data collection and analysis, decision to publish, or preparation of the manuscript?<br><br>Did you receive funding for this work?	The author(s) received no specific funding for this work.
**Competing Interests**<br><br>Use the instructions below to enter a competing interest statement for this submission. On behalf of all authors, disclose any competing interests that could be perceived to bias this work—acknowledging all financial support and any other relevant financial or non-financial competing interests.<br><br>This statement is required for submission and **will appear in the published article** if the submission is accepted. Please make sure it is accurate and that any funding sources listed in your Funding Information later in the submission form are also declared in your Financial Disclosure statement.	The authors have declared that no competing interests exist

Powered by Editorial Manager® and ProduXion Manager® from Aries Systems Corporation

View published research articles from *PLOS ONE* for specific examples.<br><br>**NO authors have competing interests**<br><br>Enter: *The authors have declared that no competing interests exist.*<br><br>**Authors with competing interests**<br><br>Enter competing interest details beginning with this statement:<br><br>*I have read the journal's policy and the authors of this manuscript have the following competing interests: [insert competing interests here]*<br><br>* typeset	
**Ethics Statement**<br><br>Enter an ethics statement for this submission. This statement is required if the study involved:<br><br>• Human participants<br>• Human specimens or tissue<br>• Vertebrate animals or cephalopods<br>• Vertebrate embryos or tissues<br>• Field research<br><br>Write "N/A" if the submission does not require an ethics statement.<br><br>General guidance is provided below. Consult the submission guidelines for detailed instructions. **Make sure that all information entered here is included in the Methods section of the manuscript.**	Approval was given to conduct this study by the Faculty of Health Sciences Ethics Committee, aproval number 309/2023. Waiver for informed consent was granted because of the retrospective nature of the study.<br>DAta were analysed anornymously.

Powered by Editorial Manager® and ProduXion Manager® from Aries Systems Corporation

**Format for specific study types**<br><br>**Human Subject Research (involving human participants and/or tissue)**<br>• Give the name of the institutional review board or ethics committee that approved the study<br>• Include the approval number and/or a statement indicating approval of this research<br>• Indicate the form of consent obtained (written/oral) or the reason that consent was not obtained (e.g. the data were analyzed anonymously)<br><br>**Animal Research (involving vertebrate animals, embryos or tissues)**<br>• Provide the name of the Institutional Animal Care and Use Committee (IACUC) or other relevant ethics board that reviewed the study protocol, and indicate whether they approved this research or granted a formal waiver of ethical approval<br>• Include an approval number if one was obtained<br>• If the study involved *non-human primates*, add *additional details* about animal welfare and steps taken to ameliorate suffering<br>• If anesthesia, euthanasia, or any kind of animal sacrifice is part of the study, include briefly which substances and/or methods were applied<br><br>**Field Research**<br><br>Include the following details if this study involves the collection of plant, animal, or other materials from a natural setting:<br>• Field permit number<br>• Name of the institution or relevant body that granted permission	
**Data Availability**<br><br>Authors are required to make all data underlying the findings described fully available, without restriction, and from the time of publication. PLOS allows rare exceptions to address legal and ethical concerns. See the PLOS Data Policy and FAQ for detailed information.	Yes - all data are fully available without restriction

Powered by Editorial Manager® and ProduXion Manager® from Aries Systems Corporation

A Data Availability Statement describing where the data can be found is required at submission. Your answers to this question constitute the Data Availability Statement and **will be published in the article**, if accepted.<br><br>**Important:** Stating 'data available on request from the author' is not sufficient. If your data are only available upon request, select 'No' for the first question and explain your exceptional situation in the text box.<br><br>Do the authors confirm that all data underlying the findings described in their manuscript are fully available without restriction?	
**Describe where the data may be found in full sentences. If you are copying our sample text, replace any instances of XXX with the appropriate details.**<br><br>• If the data are **held or will be held in a public repository**, include URLs, accession numbers or DOIs. If this information will only be available after acceptance, indicate this by ticking the box below. For example: *All XXX files are available from the XXX database (accession number(s) XXX, XXX.).*<br>• If the data are all contained **within the manuscript and/or Supporting Information files**, enter the following: *All relevant data are within the manuscript and its Supporting Information files.*<br>• If neither of these applies but you are able to provide **details of access elsewhere**, with or without limitations, please do so. For example:<br><br>*Data cannot be shared publicly because of [XXX]. Data are available from the XXX Institutional Data Access / Ethics Committee (contact via XXX) for researchers who meet the criteria for access to confidential data.*<br><br>*The data underlying the results presented in the study are available from (include the name of the third party*	All relevant data are within the manuscript and its Supporting Information files.

and contact information or URL).<br>• This text is appropriate if the data are owned by a third party and authors do not have permission to share the data.<br><br>* typeset	
Additional data availability information:	

Manuscript

Click here to access/download;Manuscript;Vulvar Retrospective manuscript PLos ONE Nov 2024.docx

Clinico-pathological and treatment characteristics of HIV and non-HIV related vulvar cancers: Analysis of a South African cohort

Adekunle Emmanuel Sajo[1*], Edwin Mnisi E[1,3], Sheynaz Bassa[4,5], Cathy Visser[1], Piet Becker[6], Greta Dreyer[1,2]

1. Department of Obstetrics and Gynaecology, Gynaecologic Oncology Unit, University of Pretoria, Pretoria, South Africa
2. Department of Obstetrics and Gynaecology, Gynaecologic Oncology Unit, Steve Biko Academic Hospital, Pretoria, South Africa
3. Department of Obstetrics and Gynaecology, Kalafong Provincial Tertiary Hospital, Pretoria, South Africa
4. Department of Radiation Oncology, University of Pretoria, Pretoria, South Africa
5. Department of Radiation Oncology, Steve Biko Academic Hospital, Pretoria, South Africa
6. Biostatistics Unit, South African Medical Research Council, South Africa

*Corresponding author

E-mail: adekunlesajo@yahoo.com (AE)

Abstract

Objective

The main objective of this study is to review the clinical, pathological and treatment characteristics of vulvar cancer patients presenting to Steve Biko Academic Hospital. This includes patients who have received surgery and/or radiotherapy as part of their treatment.

Material and Methods

A retrospective review of clinical case records of patients presenting to the Gynaecology Oncology and Radiation Oncology departments of the hospital. The clinical, pathology and radiotherapy treatment records of patients presenting between January 2012 and December 2022 were reviewed for this study

Results

317 vulvar cancer patients between 2012 and 2022 were included in the analysis. The average age was 45.1±12.7. Forty percent of the participants were younger than 40 years. More than 75% of them were women living with HIV and were all on antiretroviral treatment. The average age of those who were HIV positive was 20 years lower than their HIV negative counterparts, p <0.0001. Their mean haemoglobin (Hb) at presentation was 10.7g/dL. Squamous cell carcinoma was the most common histological type in 96.5% of cases. Sixty four percent of the cases presented in advanced stage. About 48% of those who had primary radiation

received curative doses. The median time to completion of radiotherapy treatment was higher among those who received primary radiotherapy as compared to those who received adjuvant treatment, 51.5 vs 46 days, p 0.039. The annual average age decreased from 56 years to as low as 40 years, a mean difference of 16 years, p 0.012.

Conclusion

It is striking that vulvar cancer is no longer a disease of elderly women. Its incidence is now high among women below 50 years. The study also showed an upward trend in the number of vulvar cancer cases in contrast to the declining average age at diagnosis. There is need for more vulvo-perineal surveillance of HIV positive women to detect early stage of vulvar cancers.

Keywords: Vulvar cancer, HIV, treatment

Introduction

Vulvar cancer though rare, accounts for 3-5% of all gynecologic malignancies. Globally, it accounted for over 45 000 new cases and 17 000 deaths. In 2020 global cancer estimate, the age-standardized incidence rate (ASIR) and mortality was 0.9 and 0.3 respectively**.**[1] It ranks second to cervical cancer as a human papillomavirus (HPV) associated female genital tract malignancy.[1] Vulvar cancer shares common risk factors with cervical cancer such as advanced age, smoking, high risk sexual practices, HPV infection and immune suppression. Over 90% of vulvar cancers are squamous cell cancer subtypes, with a smaller percentage made up by the uncommon histological variants including sarcoma and clear cell cancer.[2-6] There is a bimodal age distribution affecting both young and older women. The predominant age group and ASIR differ between countries, with Southern (1.5) and East Africa (1.3) having a higher ASIR for young women while Western Europe, North America and Australia demonstrate higher ASIR for women over 50 years. [7-12] The incidence of vulvar cancer has however been rising among premenopausal women albeit due to the high prevalence of HPV and co-infection of Human immunodeficiency virus (HIV).

Between 1994-2013, the ASIR and age standardized mortality rate (ASMR) increased by 16.1% and 2.6% respectively in South Africa. The median age at diagnosis decreased by 18years during same period.[13] Butt and ~~coworkers~~ **colleagues** also noted that the age of their cohorts was 15years younger than the age of women with vulvar cancer in developed world.[9] Among older women and in high income countries (HICs), vulvar cancer is mostly driven by TP53 gene mutations or other molecular and genetic processes associated with chronic

dermatologic condition like lichen sclerosus.[4, 14] Most women with vulvar cancer had a long history of pruritic or sore premalignant lesions: the HPV related usual vulvar intraepithelial neoplasia (uVIN, high grade squamous intraepithelial lesion, HSIL) or the HPV independent differentiated VIN (dVIN).[3, 4, 13, 15] The HPV-related vulvar cancer accounts for 25-45% of vulvar cancer globally**.** And this pathway has been driving most of the cases of vulvar cancers among young women in Africa.[8, 13, 15, 16] Evidence from the literature showed vulvar cancers arising from the dVIN have poor oncological outcomes as compared to the HPV dependent vulvar cancers. [4, 8, 17, 18]

Surgery, ranging from simple wide local excision to radical vulvectomy with or without inguinofemoral lymphadenectomy, is the main treatment modality for early stage diseases. Radiotherapy and chemotherapy also play significant role in advanced disease and adjuvant treatment. Adjuvant therapy is often offered post-surgery to reduce the risk of recurrence in women with adverse pathological features. About 10-40% of vulvar cancers recur with majority of the recurrences occurring within 2 years of completing primary treatment.[2, 5, 16, 19]

Vulvar cancer has been largely understudied globally. Data on the demographics, pathologic distributions and outcomes of women diagnosed with vulva cancer in sub-Saharan Africa is sparse. Africa relatively has younger populations as compared to the western world. Therefore, we suspect a different demographics of women with vulvar cancer in African subregions. This review would provide some insight into the knowledge of the demographic, pathologic features and trends of vulvar cancer Africa.

Materials and Methods

This was a retrospective review of all cases of vulvar cancer between January 2012-December 2022 at the Gynaecologic Oncology Unit at Steve Biko Academic Hospital (SBAH). Data were accessed between 1st July 2023 and 28th June 2024. Ethics approval (reference: 309/2023) was obtained from the University of Pretoria Faculty of Health Sciences Research Ethics Committee. All data were extracted from the medical records obtained from the departments of Gynecology, and Radiation Oncology. These included: age, parity, comorbidities disease stage and localization, HIV related details, pathological features and management. Laboratory data was obtained via the National Health Laboratory Services (NHLS Labtrak) electronic database. The 2009 FIGO staging was used to stage all patients for uniformity.[20] Surgery comprised of complete/radical vulvectomy, wide local excision or partial /hemi-vulvectomy. The use of unilateral or bilateral inguinofemoral lymphadenectomy

was dependent on the size, depth and laterality of the lesion. Primary chemoradiation was offered to those deemed inoperable while adjuvant radiotherapy was given to those who were at high risk for recurrence after surgery. The criteria for adjuvant treatment included: positive nodes, close or involved margins,and others features as determined/set by the multidisciplinary tumour board. Stata 18 statistical package (StataCorp LP, Texas, USA) was used for data cleaning and analysis. The demographic and clinicopathologic characteristics of the participants were presented as frequencies, tables and graphs. Continuous variables were checked for normality using the skewness-Kurtosis test. Variables that were normally distributed were presented as mean ± standard deviation, while those that were not distributed normally were presented as median and interquartile range. Pearson's Chi-squared test (or Fischer's exact test when the expected cells were less than 5 in at least 25% of the cells) was used to determine the associations between binomial and categorical variables. The Student's t-test or Wilcoxon rank sum test was used to compare age, parity, Hb level, tumour size, and CD4 count across categorical variables with two levels. One-way Analysis of Variance (ANOVA) was used to compare the mean age across the years of diagnosis, followed by the Bonferroni post hoc test. The univariable and multivariable logistic regression analysis was used to assess the predictors of lymph node metastasis. Variables with p-value <0.2 were used in a stepwise regression fashion to build the final multivariable model. The Pearson's goodness of fit (Hosmer and Lemeshow's goodness of fit test) was conducted for the final multivariable model to determine the degree of fit of the data. A model to which the variables fit the data is expected to have a P-value >0.05. The AUB was also determined to assess the discriminatory value of the model. The level of statistical significance was set at p-value <0.05, confidence interval of 95% and the two–tailed test of hypothesis was assumed.

Results

The data analysis included 317 participants with vulvar cancer treated between January 2012 and December 2022. From the 404 files that were evaluated, 87 were excluded due to incomplete or poor records and those who were erroneously coded as vulvar cancer.

Demographic and clinical characteristics

The mean age of the participants was 45.1±12.7 (median age 42 (IQR 37-51). The youngest patient was 14years while the oldest was 87years (table 1).

Table 1: Demographic and Clinical Characteristics

**Variable**	**HIV neg, n=69, (21.8%)**	**HIV pos, n=248, (78.2%)**	**P value**

**Age** (years), **45.1±12.7**	**60.6±14.7**	**40.8±8.0**	**<0.0001[a]**
Below 40	3 (2.3)	125 (97.7)	<0.001[a]
40-49	11 (11.0)	89 (89.0)	
50-59	16 (36.4)	28 (63.6)	
60 and above	39 (86.7)	6 (13.3)	
**Parity, 2(IQR:1-3)**	**3 (IQR-2-3)**	**2 (IQR-1-3)**	**<0.0001[*]**
0	7 (20.6)	27 (79.4)	<0.001[c]
1-4	35 (16.0)	184 (84.0)	
≥5	14 (53.9)	12 (46.1)	
**HB (g/dl), 10.7±2.4**	**11.6±2.5**	**10.4±2.3**	**0.0009[a]**
<10	13 (12.4)	92 (87.6)	0.001[c]
10-11.9	17 (17.9)	78 (82.1)	
≥12	36 (34.3)	69 (65.7)	
**Comorbidity**			
DM	6 (60.0)	4 (40.0)	<0.001[+]
HTN	19 (59.4)	13 (40.6)	
Cardiac	2 (66.7)	1 (33.3)	
CKD	2 (66.7)	1 (33.3)	
DM & HTN	4 (57.1)	3 (42.9)	
None	35 (13.4)	226 (86.6)	
**Performance status (ECOG)**			
0-1	48 (25.4)	141 (74.6)	0.057[c]
2-4	21 (16.4)	107 (83.6)	
**Adjacent Organ involved**			
Perianal	6 (15.9)	32 (84.2)	0.37[c]
Pelvic bone	0 (0.0)	5 (100)	
Urethra	3 (20.0)	12 (80.0)	
Vagina	1 (4.5)	21 (95.5)	
**Tumour size (cm), 5.4±2.6**<br>**Range (0.5-18cm)**	4.7±1.5	5.7±2.9	0.026[a]
<2	1 (6.3)	15 (93.8)	0.004[c]
2-4	19 (38.8)	30 (61.2)	

>4		49 (19.8)	198 (80.2)	
**Follow up (months), 18(IQR:6-48), 28.5±28**		23 (IQR:9-70)	17 (IQR:6-38)	0.036[*]
**FIGO stage**	Early	32 (28.1)	82 (71.9)	0.042[c]
	Advanced	37 (18.3)	166 (81.8)	
**Histological types**	SCC (**96.5%)**	62 (20.3)	244 (79.7)	0.006[+]
	Sarcoma	4 (66.7)	2 (33.3)	
	NEC	1 (50)	1 (50)	
	Adenocarcinoma	1 (50)	1 (50)	
	Paget's disease	1 (100)	0 (0)	
**Grade**	1	6 (23.1)	20 76.9)	0.46[c]
	2 (**75.8%)**	48 (22.2)	168 (77.8)	
	3	6 (14.0)	37 (86.0)	
**Focality**	unifocal	47 (27.2)	126 (72.8)	0.011[c]
	multifocal	22 (15.3)	122 (84.7)	
**Treatment type**	Radiation, n = 114	17 (14.9)	97 (85.1)	0.003[c]
	Surgery, n = 152	45 (29.6)	107 (70.4)	
**Interval between biopsy & first clinic visit (weeks), 10.8±9.1**		**8.9±5.7**	**11.3±9.8**	**0.055**
≤6 weeks		28 (26.7	77 (73.3)	0.12[c]
≥6 weeks		38 (18.9)	163 (81.1)	
**CD4 count (cell/µL), 481.8±260**	<200		28 (12.2)	
	201-499		100 (43.9)	
	±500		100 (43.9)	
**Viral load (copies/mL**	Lower than detectable		99 (60.4)	
	Suppressed (<1000)		36 (21.9)	
	Unsuppressed (≥1000)		29 (17.7)	
**Interval between ARV initiation and cancer diagnosis**				
<10yrs			90 (71.4)	
≥10yrs			36 (28.6)	

[a]Student t test, [c]Chi-squared, [*]Mann-Whitney U test, [+]Fishers Exact

Forty percent of the participants were younger than 40 years. Over 75% of the participants were women living with HIV. The mean age of those who were HIV positive was significantly lower than those who were living with HIV, 40yrs vs 60yrs, p <0.0001. Among those who were

below 40yrs of age, over 95% of them were women living with HIV while those who were above 60yrs of age were those without HIV. Those living with HIV were statistically significantly younger than those who were HIV negative, p <0.001. The mean haemoglobin (Hb) at first presentation was 10.7g/dL. The mean Hb was significantly lower among those who were HIV positive as compared to those who were HIV negative, 10.4g/dL vs 11.6g/dL, p 0.0009. The proportion of those who presented with Hb less than 10g/dL was significantly higher among those who were living with HIV as compared to the HIV negative counterparts, 87.6% vs 12.4%, p <0.001. Eighty two percent of the participants did not have other medical comorbidity such as diabetes, hypertension or cardiorenal diseases. There was no statistically significantly difference in the performance status across HIV category, p 0.057. The mean tumour size was 5.4cm with a range of 0.5-18cm. Those who were HIV positive had significantly larger tumour size as compared to those without HIV, 5.7cm vs 4.7cm, p 0.026. Squamous cell carcinoma was the most common histological type, accounting for 96.5%. Sixty four percent of the cases presented in advanced stage (stage III and IV). Among those who presented in late stage, 81% of them were women living with HIV. About 55% of the disease were unifocal. A greater proportion (57%) of the patients had surgery as the first modality of treatment. The mean time interval between biopsy and first presentation at the gynaecologic oncology outpatient was 10.8 weeks and HIV status did not have significant influence on this, p 0.055. Among those who were HIV positive, the mean CD4 count was 481.8±260 cell/μL while 82.3% of them either had lower than detectable or suppressed viral load. Majority of them (71.4%) were on antiretroviral therapy for less than 10yrs.

Pathological characteristics of patients who had surgery

The histopathologic features of those who had surgery are shown in table 2.

Table 2: Histopathological characteristics of surgical specimens

**Features**		N=152(%)
**Tumour size**		5.0 ±1.9
≤4cm		57(37.5)
4.1-6.0cm		69 (45.4)
6.1-8.0cm		20 (13.1)
8.1-10cm		3 (2.0)
>10cm		3 (2.0)
**focality**	Unifocal	111 (73.0)
	Multifocal	41 (27.0)

**Histologic type**		
SCC		147 (96.7)
Sarcomas		3 (1.9)
Adenocarcinoma		1 (1.0)
Paget's disease		1 (1.0)
**Grade**	1	15 (10.9)
	2	107 (77.5)
	3	16 (11.6)
**Lymph node dissection**		
ipsilateral		3 (2.0)
Bilateral		120 (81.2)
Not performed		29 (16.8)
**Number removed**		14.9±6.5, range: 2-36
<12		40(32.5)
≥12		83(67.5)
**Nodal status**	Negative	79 (64.2)
	Positive	44 (35.8)
**Number of positive nodes**		2 (IQR 1-3)
1		15 (34.1)
2		8 (18.2)
≥3		21(47.7)
**Extracapsular spread**	No	29 (65.9)
	Yes	15 (34.1)
**Surgical margin (mm)**		
<3		31 (20.4)
3-4		38 (25.0)
5-7		29 (19.1)
≥8		54 (35.5)
**Depth of infiltration (mm)**		
≤1mm		17 (15.7)
2-3mm		23 (21.3)
>3mm		68 (63.0)
**VIN present at margin**	No	77 (51.7
	Yes	72 (48.3)
**LVS**	No	47 (30.9)

	Yes	10 (6.6)
	Not reported	95 (62.5)
**Perineural involvement**	No	26 (17.1)
	Yes	4 (2.6)
	Not reported	122 (80.3

The average tumour size was 5.0cm in the largest diameter. About two-thirds (62.5%) of the tumours were larger than 4cm in the greatest dimensions. Just like in the general histological type, 96.7% of the histological type were SCC while grade 2 was the most common (77.5%) tumour differentiation. Among those with sarcomas, 2 of them were younger than 18 years with histological sub-type of Rhabdomyosarcoma. Only about a fourth of the disease involved more than one site. More than 80% of them had bilateral inguinofemoral lymphadenectomy. Among those who had nodal dissection, the mean number of nodes removed was 15. About a third of the patients had positive nodes with about half of them having 3 or more positive nodes. Among 44 patients who had positive node, 34.1% of the nodes showed extracapsular spread. Thirty five percent of the surgical free margins were 8mm or more while vulvar intraepithelial neoplasia (HSIL) was present at the surgical margin in about half of the surgical specimens. Among those reported with depth of infiltration, 63% (68/108) of the tumour infiltrated to a depth of 3mm or more.

Treatment modalities

Of the 152 patients who had surgery, about 70% had radical vulvectomy, table 3.

Table 3: **Characteristics of treatment modality**

**Primary treatment received**		
**Surgery**		**N = 152, (%)**
Partial vulvectomy		**32 (21.0)**
Radical vulvectomy		**105 (69.1)**
Wide local excision		**15 (9.9)**
**Interval between first visit and surgery** (weeks)		**4 (IQR:3-11)**
**Radiotherapy (Primary)**		**N = 165, (%)**
	Treated	**114 (69.1)**
	Not treated	**51 (30.9)**
**Treated**	Curative/radical	**54 (47.4)**
	Palliative	**60 (52.6)**

**Adjuvant RT**	Given	**29 (42.7)**	
	Not given	**39 (57.3)**	
**Interval between Referral and start of RT** (weeks)			
**18.9±11**, 20 (IQR:9-29)	**primary**	**Adjuvant**	**P value**
	20 (IQR:9-30)	16 (7-20)	0.017*
**Radiotherapy completion time (days)**			
	**Standard ≤56 days**	**Prolonged >56 days**	**P value**
48.5 (IQR:35.5-64)	51.5 (IQR:40-70)	46 (IQR 25-56)	0.039*
**primary**	31 (57.4)	23 (42.6)	0.18[C]
**Adjuvant**	21 (72.4)	8 (27.6)	

[c]Chi-squared, *Mann-Whitney U test

Only about 10% of the disease were amenable to wide local excision. The median time interval between Outpatient clinic presentation and surgery was 4 weeks (IQR:3-11). Of the 165 patients who were referred for primary radiotherapy, about 70% of them received treatment. Those who were not treated were either too sick to receive treatment or failed to present to the radiotherapy department. Forty seven percent of those who were treated received curative/radical doses of radiation, with or without chemotherapy while 52.6% of them received palliative radiation due to their advanced incurable disease. Among the 68 patients who were referred for adjuvant radiotherapy, only 42.7% received treatment. Those who did not receive adjuvant treatment either refused to go after surgery or presented at a later time when adjuvant radiotherapy was no longer beneficial. The average time interval between referral from gynaecologic oncology clinic and commencement of radiotherapy treatment was 18.9±11 weeks. This interval was statistically significantly lower for those who received adjuvant therapy as compared to those who had primary radiotherapy, 16 vs 20 weeks, p 0.017. The median time to completion of radiotherapy treatment was 48.5 days (IQR: 35.5-64). This was statistically significantly higher among those who received primary radiotherapy as compared to those who received adjuvant treatment, 51.5 vs 46 days, p 0.039. Patients who had primary or adjuvant radiotherapy did not significantly differ in either standard radiotherapy duration (≤56days) or prolonged duration of radiotherapy (>56 days), p 0.18.

Trends of vulvar cancer

Figure 1 showed the absolute number of vulvar cancers per year increased from 10 in 2012 to 33 in 2022.

Fig. 1: Trends of patient age and number of vulvar cancer cases per year

There was a significant variation in the number of cases across these years. The average age per year decreased from 56 years to as low as 40 years with a mean difference of 16 years, p 0.012. After Bonferroni post-hoc test, the difference lied between 2012 and 2018 year of diagnosis. The mean age of the patients has been reducing in a linear fashion since 2012 while the number of vulvar cases has been rising in an exponential manner since the same year. The trend for both the number of cases and the mean age of patients were relatively flat between 2015 and 2016 years of diagnosis. When the years of diagnosis were halved, the mean age at diagnosis in the first half (2012-2016) was significantly higher than that of the later half, 46 years vs 44 years, p 0.001. In fig.2, the proportion of patients diagnosed with vulvar cancer who were living with HIV steadily increased significantly per year over the periods of the study, from 20% in 2012 to reaching the highest (96.4%) in 2021, p 0.001.

Fig. 2: Distribution of HIV status among the patients

There was no difference in the stage of disease at presentation across the study years, p 0.45. There was also no difference in the pattern of disease stage between the first and second half of the years of diagnosis, p 0.53. The number of cervical cancer cases was seen to be reducing relative to the increasing number of vulvar cancers during the study period (fig. 3).

Fig.3 Distribution of cervical and vulvar cancers

Predictors of nodal metastasis among surgical group

From table 4, the univariate logistic regression analysis showed lymphovascular space involvement and haemoglobin less than 10g/dl were individually predictive of lymph node metastasis among those who had surgical treatment.

Table 4: Univariate and Multivariate Logistic Regression for the predictors of nodal metastasis among the operated patients

**Variables**	**OR**	**95% CI**	**p value**	**Adj OR**	**95% CI**	**p value**
**Age**						
50yrs and above	1.00	reference	reference	1.00	reference	reference
Below 50yrs	1.17	0.54-2.58	0.69			
**Tumour size (cm)**	Coef. 0.27	0.05-0.48	0.015*			
<4	1.00	reference	reference	1.00	reference	reference
>4	2.11	0.95-4.70	0.06	2.9	0.73-11.3	0.13

**HIV status**						
Negative	1.00	reference	reference	1.00	reference	reference
Positive	1.67	0.71-3.91	0.24	1.15	0.33-3.97	0.83
**Focality**						
Unifocal	1.00	reference	reference	1.00	reference	reference
multifocal	1.47	0.65-3.34	0.35	-	-	-
**Duration of ARV use**						
<10yrs	1.00	reference	reference	1.00	reference	reference
10yrs or more	1.81	0.47-6.97	0.39	-	-	-
**CD4 count (cells/µL)**						
<200	1.00	reference	reference	1.00	reference	reference
200-499	0.57	0.12-2.71	0.48	-	-	-
≥500	0.64	0.14-2.93	0.57	-	-	-
**Viral load**						
Lower than detectable	1.00	reference	reference	1.00	reference	reference
Suppressed	2.7	0.70-10.65	0.15	-	-	-
Unsuppressed (≥1000)	0.39	0.07-2.04	0.26	-	-	-
Haemoglobin (g/dl)						
≥12	1.00	reference	reference	1.00	reference	reference
10-11.9	1.87	0.78-4.50	0.16	2.26	0.57-8.95	0.24
<10	4.7	1.6-13.4	0.004*	5.0	1.1-18.4	0.036*
**Lymphovascular space**						
No	1.00	reference	reference	1.00	reference	reference
Yes	7.0	1.4-35.4	0.019*	1.85	0.24-14.2	0.55
**Perineural involvement**						
No	1.00	reference	reference	1.00	reference	reference
Yes	1.87	-0.59 - 4.33	0.14	-	-	-
**Tumour Grade**						
Good	1.00	reference	reference	1.00	reference	reference
Poor	3.05	0.99-9.33	0.05	5.1	1.00-25.6	0.049*
**Depth of Infiltration (mm)**						

≤1mm	1.00	reference	reference	1.00	reference	reference
2-3mm	1.71	0.15-19.4	0.66	1.46	0.09-22.6	0.79
>3mm	5.9	0.69-50.1	0.11	4.94	0.45-54.6	0.19

OR= Odd ratio, Adj. OR = adjusted odd ratio**,** CI: 95% Confidence interval, *significance

Lymphovascular space involvement had seven folds increased odds of predicting lymph node metastasis in univariate analysis. Tumour size of greater than 4cm and poor tumour grade showed trends towards significance in predicting lymph node metastasis, p 0.06 vs p 0.05 respectively. After adjusting for confounding variables such as tumour size, HIV status, lymphovascular space involvement and depth of invasion, poor tumour grade and haemoglobin levels of less than 10g/dl had 5 folds odds of predicting lymph node metastasis (a marker of advanced disease), p 0.049 vs 0.036 respectively. The Hosmer-Lemeshow test showed that the post estimation p-value was 0.07. Since this p-value was greater than 0.05, this means that our model fits the data reasonably well. The area under the curve of the receiver operating characteristic was 0.77, which suggested that the model was fairly predictive of inguinofemoral nodal metastasis.

Discussion

Our study found that the mean age of vulvar cancer was 45years and a greater proportion of the women were younger than 50years. While the mean age at diagnosis has been decreasing, the number of vulvar cancer cases per year has been rising significantly in the last decade of the study. More than three-quarters of the patients were women living with human immunodeficiency virus (HIV), one of the highest ever documented from a single institution in the literature. Those who were HIV positive were, on the average, 20 years younger than the HIV negative population. Squamous cell carcinoma was the most common histological type which is similar in proportion to what had been described in other studies.

In southern Africa, vulvar cancer is fast becoming a non-rare disease entity in view of the rising incidence especially among the younger women. Even though the age at diagnosis of vulvar cancer has been reducing globally, the average age of women with vulvar cancers in our population has significantly moved downwards away from the 7th and 8th decades benchmark quoted in many literature as the onset of vulvar cancer.[5, 21-24] The mean age of 65 years reported in a Tunisian study was closer to the average age of above 60 years quoted in most high-income countries that are predominantly of white and miscegenetic descents (Kehila 2017).[25] But

similar studies by Loggenberg and Butt et al in the Western Cape of South Africa, which is predominantly of white and coloured races, reported average ages of 52 and 54years respectively.[8, 9] In Asia, Europe and the USA, the average age at diagnosis ranged between 65 and 72 years.[22, 26-29] The reported mean age of 45 years in this study is similar to the average ages of 42 and 45 years reported in studies from Botswana and Mozambique which are neighbouring countries to South Africa.[29, 30] The average age of vulvar cancer appears to have stabilized between 40 and 45years in our institution. A 12-year review of cases of advanced vulvar cancer by Mnisi et al reported a median age of 43years.[31] Aside the methodological differences between our study and Mnisi et al that reported on data between 2001 and 2013 from two academic hospitals, the two years overlap of the study period did not affect the age comparison. It is striking that 40% of our study population were younger than 40years. Chikandiwa et al reported an increase of 1.7 per 100 000 in age specific incidence rate of vulvar cancer among women between 30-39 years in the 2009-2012 period as compared to an increase of 0.1 in the period 1994-1998.[13] This age group is significant both to reproduction and workforce of the country. In contrast to our findings, studies from West African countries reported average age at vulvar cancer diagnosis ranging between 48 and 61 years.[32, 33]

Human immunodeficiency virus is prevalent in most sub-Saharan African countries. South Africa is one of the countries in the world with the highest proportion of women living with HIV. The aetiological factors associated with vulvar cancer is somewhat geographical. It is reported that most vulvar cancers in Africa are associated with HPV as against the HPV-independent vulvar cancers in high income countries.[8, 9, 13, 29] HPV and HIV play synergistic role in the dysplastic changes within the epithelial lining of the lower anogenital tract**.** The rising incidence of vulvar cancer among younger age women is therefore related to the increase in HIV and HPV coinfections in South Africa.[8, 9, 13, 29] Over 75% of the women in our study were HIV positive. This is one of the highest proportions ever documented in the literature. It is also higher than the prevalence of 40% reported in our institution a decade earlier than the period covered in our study.[31] In Botswana studies, the proportions of HIV positivity among the cohorts of women with vulvar cancer ranged between 56.9 and 89%.[30, 34] The 25% national prevalence of HIV among Batswana women in the reproductive age in these studies was similar to the statistics from South Africa.[35] Aside the population difference, both countries share similar demographics, hence, not surprising that the mean age of 40years among the HIV positive women in our study is similar to the median age of 41years reported in Botswana among those living with HIV who had vulvar cancer. In our cohorts, the proportion of HIV in

those younger than 50years was 93.8%. All the WLWH in the present study were on ARVs with a median interval between the commencement of ARV and vulvar cancer diagnosis of 6 years. This was relatively similar to the interval of 9 years reported by MacDuffie and coworkers. Most of the patients were relatively immunocompetent, mean $CD4^+$ of 481 cells/µL as well as being virally suppressed. Hence, being immunocompetent and virally suppressed from chronic use of ARV might not positively influence the initiation and progression of HPV related vulvar dysplasia or there might be another pathway for the carcinogenesis of vulvar cancer in WLWH.

Furthermore, apart from the influence of HIV on the age at diagnosis of vulvar cancer, it also impacts on the clinicopathologic characteristics of women with vulvar cancer. Not only was the mean haemoglobin level lower, the tumour size was significantly bigger in WLWH as compared to their counterparts in our study. The lower haemoglobin could be due to persistent bleeding from larger tumours, chronic ARV use and effect of HIV on haemopoiesis. MacDuffie et al did not find any impact of HIV on the haemoglobin levels of patients with vulvar cancer. Most other literature did not describe the impacts of HIV on the clinicopathologic features of vulvar cancer. Hence, a need for a prospective ongoing study in our institution to describe this impact.

The most prevalent histological type in our study was squamous cells carcinoma (SCC) which accounted for over 95%. This agrees with the findings in most literature where SCC was reported as the commonest histological type.[2, 25] Vulvar squamous cell carcinoma is dichotomous. It can either be HPV dependent or HPV non-dependent, each arising from two different premalignant lesions. Often times, the HPV dependent is non-keratinizing while epithelial keratinization of vulvar tumours is associated with HPV independent long-standing lichen sclerosus or differentiated vulvar intraepithelial neoplasia.[6, 29, 36] The most common vulvar SCC, basaloid and warty types, are mostly driven by high risk HPV infection and has been found to have better prognosis.[6] Tumour size plays a significant role in vulvar cancer management and risk stratification. Surgical modality for larger tumours is associated with significant morbidity, compromise of vital contiguous structures, use of flap to close defects and risk of inadequate free margins. About eighty percent (79.2%) of our study population presented with tumour larger than 4cm. The left vulva was the most affected site while the perianal area was the most involved contiguous site. This was different from the report of de Melo Maia et al where the vaginal was the most affected adjacent structure in 15% of cases.[21] Their finding of unilateral localization of tumour was also similar in our report. In a Northern

Africa study, the average tumour size was less than 4cm while FIGO III was the highest stage at presentation.[25] Unlike in America and other HICs, patients often present in advanced stage disease in Africa and other low and middle income countries (LMIC). Two-thirds of the patients seen during the study period presented in either stage III or IV. This often reflects poor access to healthcare, poor referral system, poverty, misdiagnosis, and insufficient health education and awareness on the parts of the patients. Oncological services in South Africa, far better than in most other African countries, are regionalized in cities. Hence, patients often need to be transported from remote areas to access these services usually at an advanced stage. A reflection of these challenges was noticed in our study where the average time between biopsy at local clinics and presentation at the regional specialty clinic was 10 weeks while the average time to commencement of radiotherapy was 18 weeks from the time of referral from the gynaecology specialty clinic.

Surgery, with variable modalities, is the standard form of treatment for vulvar cancer. Radiotherapy with or without chemotherapy is offered as the primary treatment for advanced disease and as adjuvant treatment for surgically treated patients who are at increased risk of disease recurrence. The general recommendation for a tumour not more than 2cm is wide local excision with or without inguinofemoral lymphadenectomy depending on the depth of stromal invasion.[2, 19, 21, 25] Of the 266 patients who were treated, 57% had surgery. This was in contrast to the study from Brazil where over 85% of their patients had surgery which might be related to the early stage presentation reported in majority of their cohorts.[21] Radical vulvectomy was the most frequently performed surgical modality in our study because the greater proportion of the surgical group presented with larger tumours. In Kehila et al, all their patients were surgically managed as none of them presented in stage IV.[25] About 70% and 42% of our study population were able to receive primary and adjuvant radiotherapy treatment respectively. The identifiable factors responsible for these proportions include prolonged appointment waiting time to access radiotherapy services, poor clinical state, prolonged interval after primary surgery, refusal to show for treatment and loss to follow up. There is paucity of radiotherapy services across Africa which often results in an increased burden of referrals on the limited services. The median time to completion of primary radiotherapy for locally advanced vulvar cancer in our study was 52 days. The completion of radiotherapy within the scheduled time interval is a determinant that is associated with improvement in oncological survivals. This improvement in survival has only been demonstrated in locally advanced cervical cancer.[28, 37] In a study by Song et al, a completion time beyond 56 days was associated with increased risk

of pelvic recurrence in cervical cancer.[37] However, this has not been well demonstrated in vulvar cancer even though there are similarities between the two cancers in terms of carcinogenesis, behavior and treatments.

Lymph node metastasis is the most important independent negative prognostic predictors in vulvar cancer. There are several potential predictors of lymph node metastasis which define the stage and treatment of the disease. These include tumour size, poor grade, aggressive histological types, performance status, tumour localization, deep stromal invasion, lymphovascular space and perineural invasion.[2, 22, 38, 39] Only poor tumour differentiation was predictive of lymph node metastasis in our study after controlling for confounders. Reports from other studies have been inconsistent in the factors defining nodal disease prediction.[40-42] In a Danish study of 388 vulvar SCC, increasing tumour size, tumour close to the clitoris and poor grade were all significantly associated with increased risk of inguinofemoral nodal metastasis.[38] The 35% lymph node positivity in our surgical cohorts was consistent with the findings of 28-38% nodal positivity in many other literatures.[22, 38, 43] However, this was higher than the prevalence of 16% of positive nodes among 15 000 vulvar SCC from the USA National Cancer Database.[41] The low proportion of nodal positivity in this USA study might be related to the higher proportion of early stage disease among their cohorts.

Globally in the past few decades, vulvar cancer cases have been showing upward trends. Likewise, the average age at the onset of diagnosis has also been reducing. In our study, the absolute number of vulvar cancers increased from 10 in 2012 to 33 in 2022 with significant variation between these years. Relative to 2012, our findings showed an exponential upward trend in the number of cases across the years of study while the average age at diagnosis showed a linear downward trend. This trend was pronounced between 2012 and 2018 as the average age decreased by 16 years during this interval. This is similar to a decline of 18 years reported over 8 years period by Chikandiwa et al.[13] The incidence of vulvar cancer declined as the age of women increased beyond 50 years. This suggests an increase in HPV related vulvar cancers rather than the HPV independent types. However, the decline in trend of cervical cancer cases during the study period might be due to the increase awareness and uptake of cervical cancer screening especially among WLWH. While Schuurman et al found a change in FIGO stage distribution especially with increase in stages I and III diseases over their study years, our finding did not show a trend away from the usual late disease stage at diagnosis.[12] The poor help-seeking behavior, delay in referral by health professionals and prolonged appointments might be contributory to the lack of trend in stage at presentation. We also demonstrated a trend

towards the diagnosis of vulvar cancer among young HIV positive women. However, the trend of HIV positivity among those with vulvar cancer continued to increase to the highest proportion of 96% in 2021**.** In recent times, we can almost predict with certainty that a woman below 50 years who present with vulvar lesions suspected to be VIN or cancer, is living with HIV.

The limitation of this study lies in the inherent drawbacks of retrospective study. Not all variables were complete for analysis. The impact of this on our findings might be limited as missing values were corrected for in the analysis. Also, the multi-file system within the institution might be the reason why some patients were reported as no show because the radiotherapy department uses different hospital numbers to code patients in their filings system. Hence, it is possible some patients were coded with different names and hospitals numbers different from the ones used during referral at the Gynaecologic Oncology Unit. But efforts were made to search the Radiotherapy Unit database using all available patients' names, hospital numbers and national identity number to reduce this limitation. The reasons why some of the known risk factors for nodal metastasis such as lymphovascular space, perineural invasion and depth of in invasion, did not reach significant levels in our study could be that some of these parameters were not uniformly reported by the pathologists.

However, the strength of our study lies in the large study population from a single academic hospital where the treatment patterns were similar. The pathology reports were centralized to the national laboratory database, making accessibility to be easy. Also, our study included 248 women that were living with HIV, the highest reported cohorts from a single institution in the literature. This provided us with insight into this special group of high-risk individuals in our population and the need to protect them.

In conclusion, it is striking that vulvar cancer is no longer a disease attributable to older aged **Remove d.**
Remove as only women get vulvar cancer
people. Its incidence is now high among women below 50 years. This study has shown some insights into why women younger than 50 years could be susceptible to vulvar cancer. Despite being immunologically competent, women living with HIV are still at an increased risk of vulvar cancer. Hence, the need for more proactive measure to screen them not just for cervical cancer but also for vulvar cancer. We recommend that women living with HIV should have at least an annual vulvo-perineal examination in addition to the regular health checks since they visit health care centers to collect the ARV medications. Public enlightenment on the signs and symptoms of early stage vulvar would help in early detection and treatment to reduce the

incidence and improve survival. The need for synchronized electronic medical records across the departments that run oncology services will help prevent missing data thereby improving clinical care and follow up. Our study is an important additional to the growing body of evidence on vulva cancer in Africa.

Acknowledgment

We acknowledged Steve Biko Academic Hospital Medical Health Records, Radiooncology and Gynaecologic clinic staff who assisted in retrieving the patient medical records and my colleagues for their supports and encouragement.

Conflict of interest

The authors declare that there were no conflicts of interest

Authors Contributions

Conceptualization:, Sajo AE, Dreyer G, Visser S, Mnisi E, Bassa S

Supervision: Dreyer G, Sajo AE, Bassa S

Validation: Dreyer G, Bassa S, Mnisi E

Data curation: Sajo AE, Cathy Visser, Bassa S

Formal analysis: Sajo AE, Becker P

Methodology: Sajo AE, Dreyer G, Becker P

Writing-Review & Editing: Sajo AE, Dreyer G, Bassa S, Visser C, Mnisi E

List of References

1. Sung H, Ferlay J, Siegel RL, Laversanne M, Soerjomataram I, Jemal A, et al. Global cancer statistics 2020: GLOBOCAN estimates of incidence and mortality worldwide for 36 cancers in 185 countries. CA Cancer J. Clin. 2021; 71(3):209-49.

2. Olawaiye AB, Cotler J, Cuello MA, Bhatla N, Okamoto A, Wilailak S, et al. FIGO staging for carcinoma of the vulva: 2021 revision. International Journal of Gynecology & Obstetrics. 2021; 155(1):43-7.

3. Faber MT, Sand FL, Albieri V, Norrild B, Kjær SK, Verdoodt F. Prevalence and type distribution of human papillomavirus in squamous cell carcinoma and intraepithelial neoplasia of the vulva. Int. J. Cancer. 2017; 141(6):1161-9.

4. Thuijs NB, van Beurden M, Bruggink AH, Steenbergen RD, Berkhof J, Bleeker MC. Vulvar intraepithelial neoplasia: incidence and long-term risk of vulvar squamous cell carcinoma. Int. J. Cancer. 2021; 148(1):90-8.

5. Pirot F, Chaltiel D, Ouldamer L, Touboul C, Raimond E, Carcopino X, et al. Patterns of first recurrence and outcomes in surgically treated women with vulvar cancer: results from FRANCOGYN study group. Journal of Gynecology Obstetrics and Human Reproduction. 2020; 49(9):101775.

6. De Sanjosé S, Alemany L, Ordi J, Tous S, Alejo M, Bigby SM, et al. Worldwide human papillomavirus genotype attribution in over 2000 cases of intraepithelial and invasive lesions of the vulva. Eur. J. Cancer. 2013; 49(16):3450-61.

7. Bray F, Laversanne M, Weiderpass E, Arbyn M. Geographic and temporal variations in the incidence of vulvar and vaginal cancers. Int. J. Cancer. 2020; 147(10):2764-71.

8. Loggenberg FE, Adams TS. A review of vulvar carcinoma at Groote Schuur hospital for the period 2002 to 2012 with particular emphasis on HPV-related disease. Southern African Journal of Gynaecological Oncology. 2020; 12(1):17-22.

9. Butt J, Botha M. Vulvar cancer is not a disease of the elderly: Treatment and outcome at a tertiary referral centre in South Africa. S. Afr. Med. J. 2017; 107(11):1000-4.

10. Zongo N, Korsaga-Somé N, Banata Gang-Ny A, Ouangré E, Zida M, Ouédraogo AS, et al. Cancer of the vulva in Burkina Faso: a hospital-based case series. Infect. Agent. Cancer. 2016; 11:1-6.

11. Eva L, Sadler L, Thompson JM, Sahota S, Fong KL, Jones RW, et al. HPV-independent and HPV-associated vulvar squamous cell carcinoma: two different cancers. International Journal of Gynecologic Cancer. 2022; 32(9)

12. Schuurman M, Van Den Einden L, Massuger LF, Kiemeney L, van der Aa MA, de Hullu JA. Trends in incidence and survival of Dutch women with vulvar squamous cell carcinoma. Eur. J. Cancer. 2013; 49(18):3872-80.

13. Chikandiwa A, Pisa PT, Sengayi M, Singh E, Delany-Moretlwe S. Patterns and trends of HPV-related cancers other than cervix in South Africa from 1994–2013. Cancer Epidemiol. 2019; 58:121-9.

14. Kortekaas KE, Bastiaannet E, van Doorn HC, van Steenwijk PJdV, Ewing-Graham PC, Creutzberg CL, et al. Vulvar cancer subclassification by HPV and p53 status results in three clinically distinct subtypes. Gynecol. Oncol. 2020; 159(3):649-56.

15. Bornstein J, Bogliatto F, Haefner HK, Stockdale CK, Preti M, Bohl TG, et al. The 2015 International Society for the Study of Vulvovaginal Disease (ISSVD) terminology of vulvar squamous intraepithelial lesions. J. Low. Genit. Tract Dis. 2016; 20(1):11-4.

16. Nooij LS, Brand FA, Gaarenstroom KN, Creutzberg CL, de Hullu JA, van Poelgeest MI. Risk factors and treatment for recurrent vulvar squamous cell carcinoma. Crit. Rev. Oncol. Hematol. 2016; 106:1-13.

17. Lebreton M, Carton I, Brousse S, Lavoué V, Body G, Levêque J, et al. Vulvar intraepithelial neoplasia: classification, epidemiology, diagnosis, and management. Journal of gynecology obstetrics and human reproduction. 2020; 49(9):101801.

18. Al-Ghamdi A, Freedman D, Miller D, Poh C, Rosin M, Zhang L, et al. Vulvar Squamous Cell Carcinoma in Young Women: A Clinicopathologic Study of 21 Cases. Gynecol. Oncol. 2002; 84(1):94. doi:10.1006/gyno.2001.6466

19. Pedrão PG, Guimarães YM, Godoy LR, Possati-Resende JC, Bovo AC, Andrade C, et al. Management of Early-Stage Vulvar Cancer. Cancers (Basel). 2022; 14(17) doi:10.3390/cancers14174184

20. Pecorelli S. Revised FIGO staging for carcinoma of the vulva, cervix, and endometrium. Int. J. Gynaecol. Obstet. 2009; 105(2):103-4. doi:10.1016/j.ijgo.2009.02.012

21. de Melo Maia B, Munhoz Cestari F, Lavorato-Rocha AM, Sant'Ana Rodrigues I, Baiocchi G, Cardoso Guimarães G, et al. Characterization of sociodemographic and clinicopathological features in Brazilian patients with vulvar squamous cell carcinoma. Gynecol. Obstet. Invest. 2013; 75(1):53-60. doi:10.1159/000343758

22. Woelber L, Mahner S, Voelker K, Zu Eulenburg C, Gieseking F, Choschzick M, et al. Clinicopathological prognostic factors and patterns of recurrence in vulvar cancer. Anticancer Res. 2009; 29(2):545-52.

23. Te Grootenhuis NC, Pouwer AW, de Bock GH, Hollema H, Bulten J, van der Zee AGJ, et al. Margin status revisited in vulvar squamous cell carcinoma. Gynecol. Oncol. 2019; 154(2):266-75. doi:10.1016/j.ygyno.2019.05.010

24. Nishio S, Matsuo K, Shibata T, Yamaguchi S, Kanao H, Takehara K, et al. Changes in the Clinicopathological Demographics of Vulvar Cancer in Japan: Increasing Oldest-Old, Stage Shifting, and Decreasing Cohort-Level Survival (†). J Clin Med. 2019; 8(12) doi:10.3390/jcm8122081

25. Kehila M, Harabi S, Mhiri R, Touhami O, Abouda HS, Khlifi A, et al. Vulvar cancer in Tunisia: Epidemiological and clinicopathological features multicentric study. J. Egypt Natl. Canc. Inst. 2017; 29(2):95-8. doi:https://doi.org/10.1016/j.jnci.2017.02.001

26. Gensthaler L, Joura EA, Alemany L, Horvat R, de Sanjosé S, Pils S. The impact of p16(ink4a) positivity in invasive vulvar cancer on disease-free and disease-specific survival, a retrospective study. Arch. Gynecol. Obstet. 2020; 301(3):753-9. doi:10.1007/s00404-020-05431-7

27. Taran FA, Pasternak J, Staebler A, Rohner A, Neis F, Engler T, et al. Tumor-Free Resection Margin Distance in the Surgical Treatment of Node-Negative Squamous Cell Cancer of the Vulva Has No Impact on Survival: Analysis of a Large Patient Cohort in a Tertiary Care Center. Cancers (Basel). 2023; 15(16) doi:10.3390/cancers15164110

28. Nguyen NT, Zhao X, Ponzini M, Wilson M, Leiserowitz G, Brooks RA. Time to completion of radiation treatment in locally advanced squamous cell carcinoma of the vulva and the impact on survival. Gynecol. Oncol. 2022; 167(2):159-66. doi:10.1016/j.ygyno.2022.09.018

29. Rakislova N, Carreras-Dieguez N, Manzotti C, Saúde O, Del Pino M, Chulo L, et al. Differential etiopathogenic features of vulvar squamous cell carcinomas in sub-Saharan Africa and Europe. Int. J. Cancer. 2023; 152(3):496-503. doi:10.1002/ijc.34314

30. MacDuffie E, Sakamuri S, Luckett R, Wang Q, Bvochara-Nsingo M, Monare B, et al. Vulvar cancer in Botswana in women with and without HIV infection: patterns of treatment and survival outcomes. Int. J. Gynecol. Cancer. 2021; 31(10):1328-34. doi:10.1136/ijgc-2021-002728

31. Mnisi F DG. Treatment-and-outcomes-of-late-stage-vulva-cancer-at-pretoria-academic-hospital-complex-south-africa. Obstetrics and Gynaecology Forum. 2019; 2(29):16-8.

32. Jedidiah DSGE. Vulva carcinoma at the university of Benin teaching hospital a ten year review, Jedidiah et al 2015. J Medicine and Biomedical Research. 2015; 14(2):129-37.

33. Okoro CAOOMAOAAEE. A Review of Vulvar and Vaginal Cancers in Ibadan, Nigeria. North American Journal of Medicine and Science. 2013; 6(2):76-81. doi:10.7156/najms.2013.060276

34. Tesfalul M, Simbiri K, Wheat CM, Motsepe D, Goldbach H, Armstrong K, et al. Oncogenic viral prevalence in invasive vulvar cancer specimens from human immunodeficiency virus-positive and -negative women in Botswana. Int. J. Gynecol. Cancer. 2014; 24(4):758-65. doi:10.1097/igc.0000000000000111

35. Africa SS. Mid-year population estimates 2021. In: Africa SS, editor. Pretoria2021.

36. Mix JM, Gopalani SV, Simko S, Saraiya M. Trends in HPV- and non-HPV-associated vulvar cancer incidence, United States, 2001-2017. Prev. Med. 2022; 164:107302. doi:10.1016/j.ypmed.2022.107302

37. Song S, Rudra S, Hasselle MD, Dorn PL, Mell LK, Mundt AJ, et al. The effect of treatment time in locally advanced cervical cancer in the era of concurrent chemoradiotherapy. Cancer. 2013; 119(2):325-31. doi:10.1002/cncr.27652

38. Schnack TH, Froeding LP, Kristensen E, Niemann I, Ørtoft G, Høgdall E, et al. Preoperative predictors of inguinal lymph node metastases in vulvar cancer - A nationwide study. Gynecol. Oncol. 2022; 165(3):420-7. doi:10.1016/j.ygyno.2022.04.009

39. Pergialiotis V, Feroussis L, Rouvali A, Liatsou E, Haidopoulos D, Rodolakis A, et al. Perineural Invasion as a Predictive Biomarker of Groin Metastases and Survival Outcomes in Vulvar Cancer: A Meta-Analysis. Cancer Invest. 2022; 40:733 - 41.

40. Long Y, Yao DS, Wei YS, Wei CH, Chen XY. Prognostic significance of perineural invasion in vulvar squamous cell carcinoma. Cancer Manag. Res. 2019; 11:4461-9. doi:10.2147/cmar.S198047

41. Cheraghlou S, Doudican NA, Criscito MC, Stevenson ML, Carucci JA. Defining and quantifying histopathologic risk factors for regional and distant metastases in a large cohort

of vulvar squamous cell carcinomas. J. Am. Acad. Dermatol. 2023; 89(5):1022-30. doi:10.1016/j.jaad.2023.03.049

42. Nahshon C, Leitao MM, Jr., Lavie O, Segev Y. Effect of known pathological risk factors on the incidence of metastatic lymph nodes and survival in early-stage vulvar cancer: SEER analysis. Int. J. Gynaecol. Obstet. 2024; 164(1):339-44. doi:10.1002/ijgo.15093

43. Klapdor R, Wölber L, Hanker L, Schmalfeldt B, Canzler U, Fehm T, et al. Predictive factors for lymph node metastases in vulvar cancer. An analysis of the AGO-CaRE-1 multicenter study. Gynecol. Oncol. 2019; 154(3):565-70. doi:10.1016/j.ygyno.2019.06.013

Click here to access/download;Figure;Fig 1 2024.tif

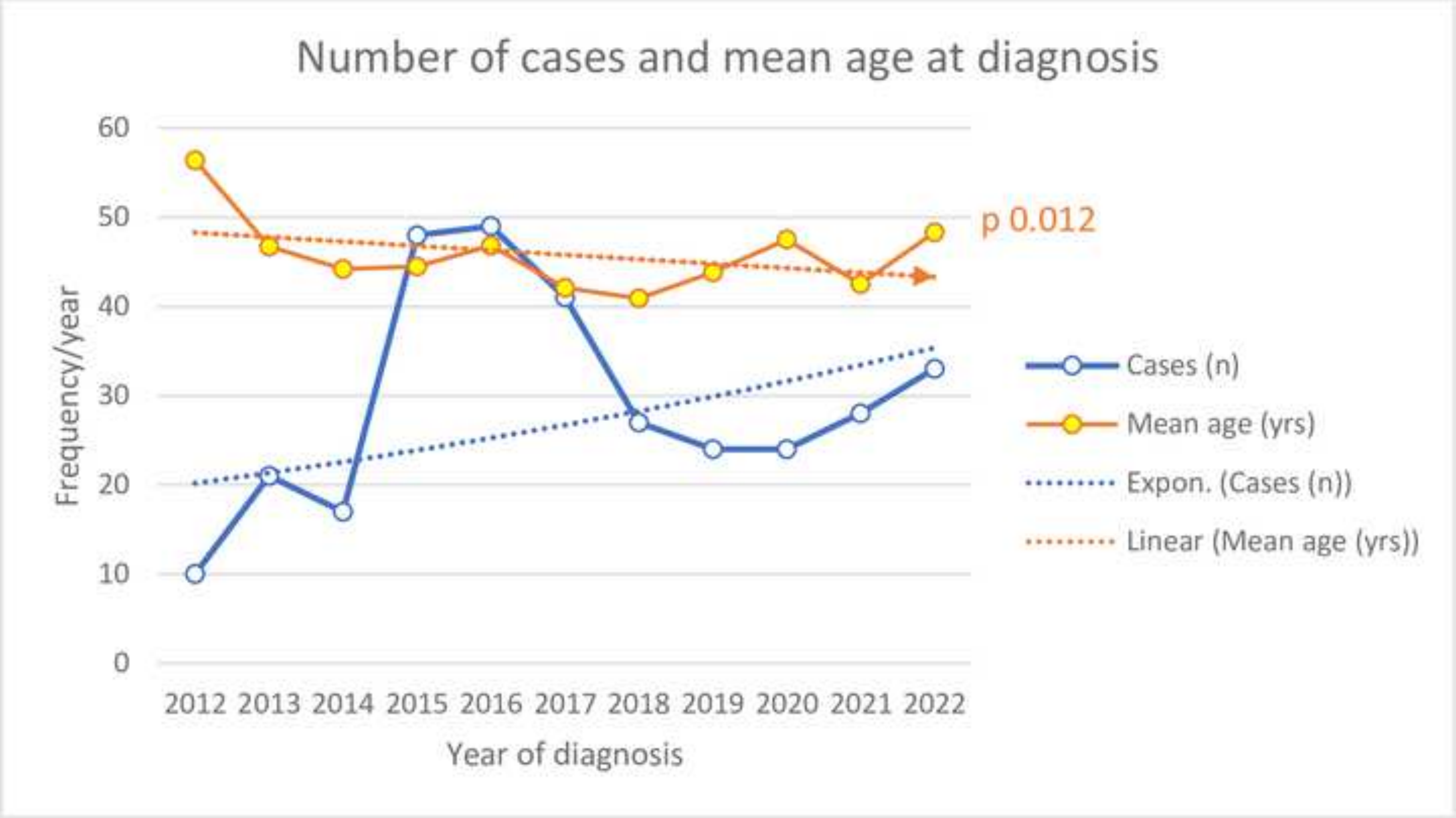

Click here to access/download;Figure;Fig 2 2024.tif

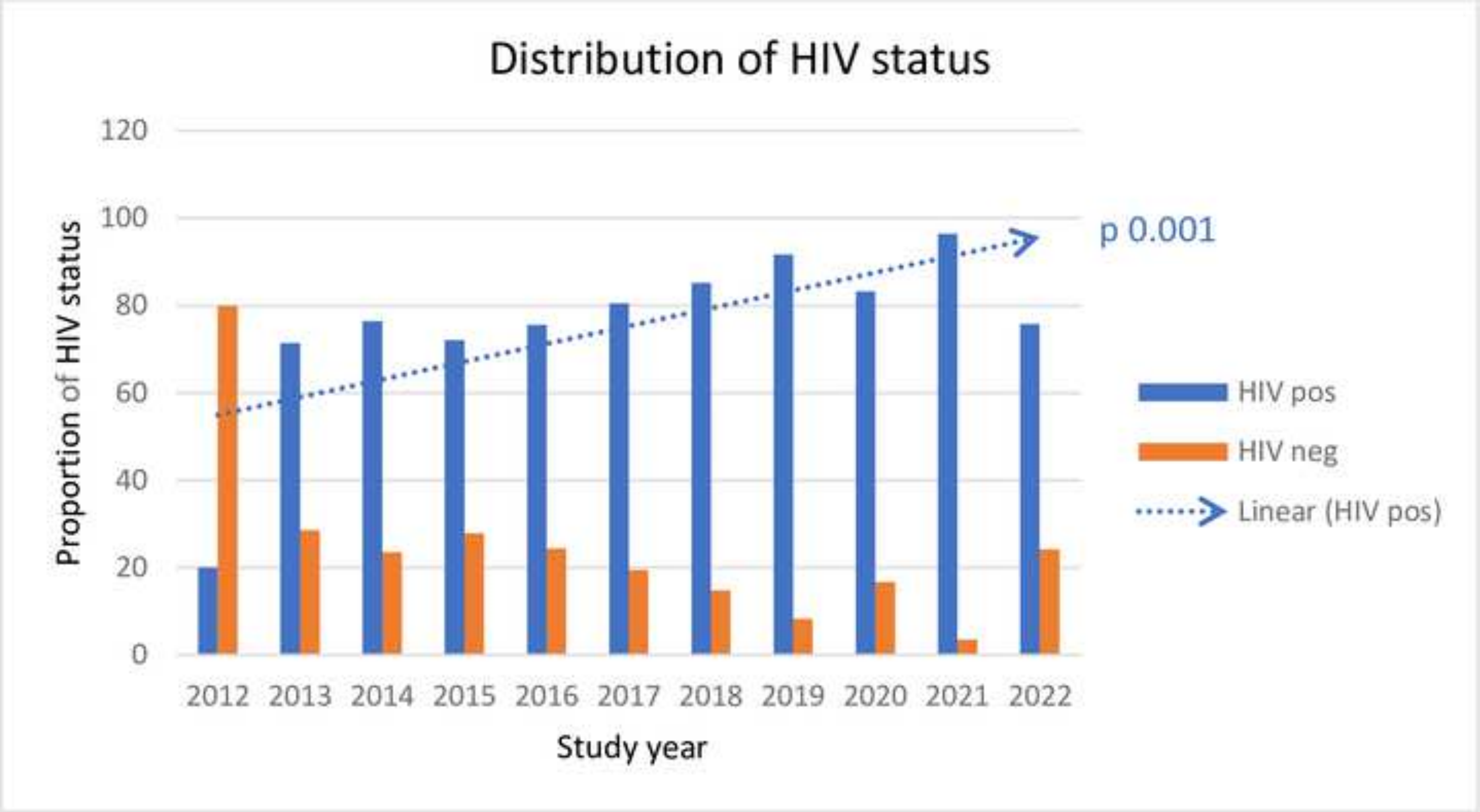

Click here to access/download;Figure;Fig 3 2024.tif

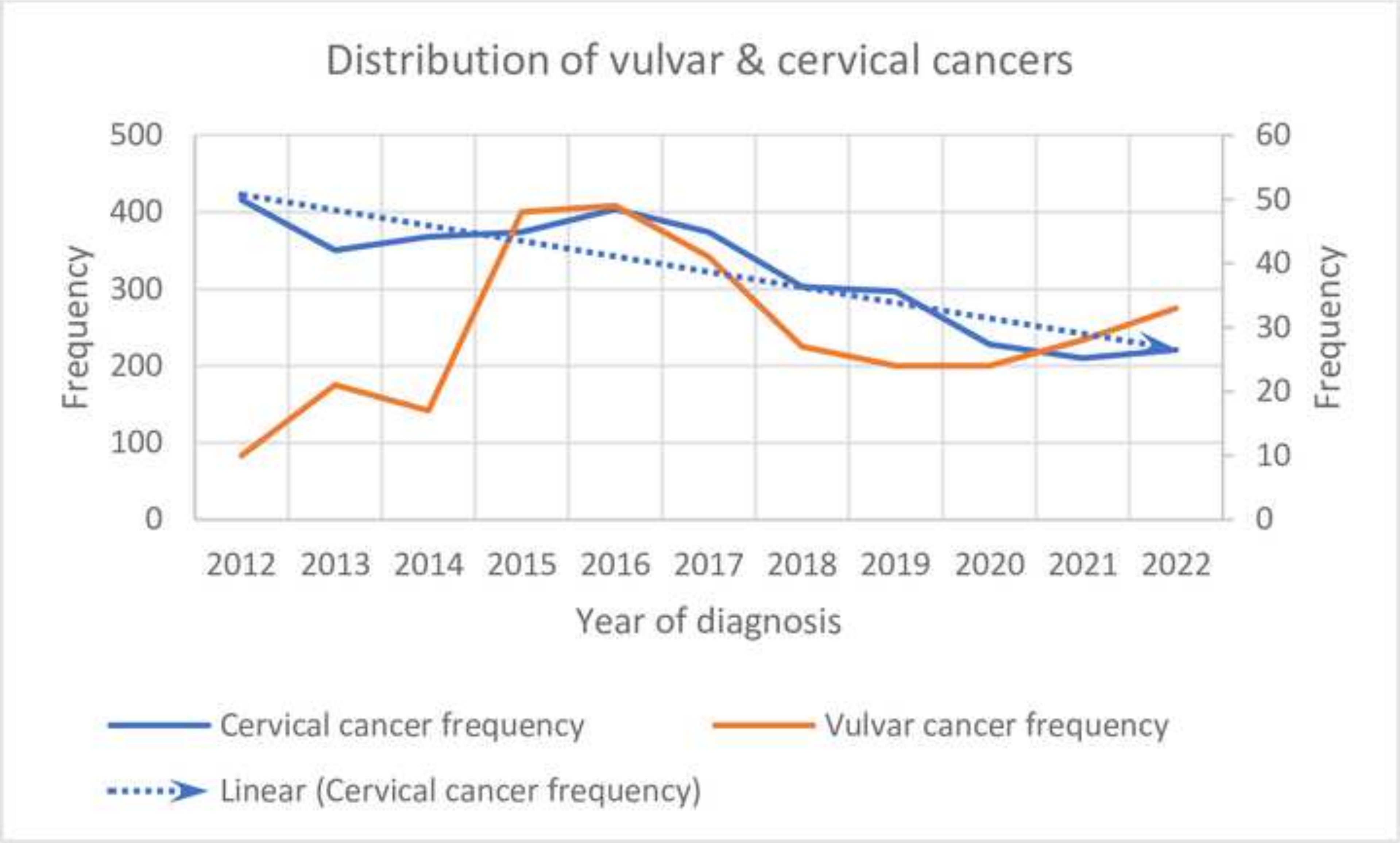

Minimal data set

Click here to access/download
Supporting Information
Excel sheet for PLos ONE manuscript Nov 5 2024.xlsx

Click here to access/download

Supporting Information

PLOSOne_Human_Subjects_Research_Checklist.docx
