# Supplementary material for: Clinico-pathological and treatment characteristics of HIV and non-HIV related vulvar cancers: Analysis of a South African cohort
Source: PLoS One. 2025 Jul 1;20(7):e0327119. doi: 10.1371/journal.pone.0327119 (PMC12212490; doi:10.1371/journal.pone.0327119)
Supplement: Attachment — Submitted filename: PlosOne Review.docx [file pone.0327119.s004.docx]

**PlosOne Review	14 May 2024**

**Clinico-pathological and treatment characteristics of HIV and non-HIV related vulvar cancers: Analysis of a South African cohort**

**Minor grammatical corrections:**

Discussion:

“Aside the population difference” Insert “from”

“Hence, a need for a prospective ongoing study in our institution to describe this impact”. Correct grammar

“The most common vulvar SCC, basaloid and warty types, are mostly driven by high risk HPV infection and has been found to have better prognosis” Correct to singular
