# Supplementary material for: Clinico-pathological and treatment characteristics of HIV and non-HIV related vulvar cancers: Analysis of a South African cohort
Source: PLoS One. 2025 Jul 1;20(7):e0327119. doi: 10.1371/journal.pone.0327119 (PMC12212490; doi:10.1371/journal.pone.0327119)
Supplement: Attachment — Submitted filename: Rebuttal May 2025.docx [file pone.0327119.s006.docx]

20^th^ May 2025

The Editor,

PLOS ONE Journal.

Dear Editor,

**REBUTTAL LETTER**

We are grateful for the thorough review of our revised manuscript by the editors and reviewers.

We have critically reviewed the comments and suggestions regarding the revised manuscript.

We addressed the corrections as follows:

**Editor’s comments**

**Please review your reference list to ensure that it is complete and correct. If you have cited papers that have been retracted, please include the rationale for doing so in the manuscript text, or remove these references and replace them with relevant current references. Any changes to the reference list should be mentioned in the rebuttal letter that accompanies your revised manuscript.**

**Response:** We critically reviewed the references again and they are complete and none has been retracted. However, reference 35 and 36 were corrected for proper citation.

**Reviewers’ comments: Minor grammatical corrections**

1. “Aside the population difference” insert from

**Response:** “from” has been inserted “Aside from the…”

2. **“Hence, a need for a prospective ongoing study in our institution to describe this impact”. Correct grammar**

**Response: “**Hence, there is a need for a prospective study in our institution to describe this impact.

3. **“The most common vulvar SCC, basaloid and warty types, are mostly driven by high risk HPV infection and has been found to have better prognosis” correct to singular**

**Response:** The most common vulvar SCC, basaloid or warty type, is mostly driven by high risk HPV infection and has been found to have better prognosis.

The corrections made are shown in the document marked tract changes and the revised document that reflects the corrections is also submitted.

Once again, we appreciate the thorough and honest review to make the manuscript a better one.

Yours sincerely,

Sajo AE (Corresponding author)
